# First Report of Extended-Spectrum β-Lactamase (*bla*_CTX-M1_) and Colistin Resistance Gene *mcr-1* in E. coli of Lineage ST648 from Cockroaches in Tunisia

**DOI:** 10.1128/spectrum.00036-21

**Published:** 2022-03-01

**Authors:** Sarrah Landolsi, Rachid Selmi, Linda Hadjadj, Asma Ben Haj Yahia, Kaouther Ben Romdhane, Lilia Messadi, Jean Marc Rolain

**Affiliations:** a Université de Tunis El Manar, Faculté des Sciences de Tunis, Tunis, Tunisia; b Université Manouba, École Nationale de Médecine Vétérinaire, Service de Microbiologie et d’Immunologie, Sidi Thabet, Tunisia; c Aix Marseille Université, IRD, APHM, MEPHI, Faculté de Médecine et de Pharmacie, Marseille, France; d IHU Méditerranée Infection, Marseille, France; Howard University

**Keywords:** cockroaches, ESBL, *Enterobacteriaceae*, ST648, Tunisia, colistin resistance, *mcr-1*

## Abstract

The emergence of multidrug-resistant bacteria has become a major problem. Cockroaches may play an important role in the spread of those bacteria between the environment and humans. This study was designed to screen extended-spectrum β-lactamase (ESBL)-producing and colistin-resistant strains and to investigate the molecular support of multidrug-resistant *Enterobacteriaceae* in the external surface and gut homogenates of cockroaches collected from different locations in Tunisia. Between July 2017 and June 2018, 144 *Enterobacteriaceae* samples were isolated from 115 trapped cockroaches (collective catering, houses, and a hospital). Antibiotic susceptibility testing was performed using the disk diffusion method. Extended-spectrum β-lactamase-encoding genes and the *mcr-1* gene were investigated by real-time PCR (RT-PCR) and standard PCR. The genetic relationship among isolates was studied with the help of multilocus sequence type (MLST) analysis. Of the 144 *Enterobacteriaceae* isolates, 22 strains exhibited a positive ESBL-screening test (73.3%), including 17 Escherichia coli isolates and 5 Klebsiella pneumoniae isolates. Among them, 9 Escherichia coli isolates were resistant to colistin, with an MIC ranging from 8 to16 μg/L, all of which harbored the *mcr-1* gene. Eight *bla*_CTX-M-15_ genes were detected; two among them were associated with *bla*_TEM-117_ and *bla*_TEM-128_, and seven *bla*_CTX-M-1_ genes were detected that also harbored the *mcr-1* gene. Genotyping analysis revealed 7 different sequence types already described in humans and animals. We report the first survey of *mcr-1* in ESBL-producing E. coli isolates from cockroaches. Our findings highlight cockroaches as a source of nosocomial infections, and they are a reservoir of colistin-resistant E. coli, which is a carrier of other additional risk genes such as *bla*_ESBL_, especially in hospitals.

**IMPORTANCE** Multidrug resistance in *Enterobacteriaceae* has become a major concern worldwide that is increasingly observed in human, animals, and also cockroaches. In our study, we found that cockroaches may play an important role as a potential vector of multidrug-resistant *Enterobacteriaceae* in the hospital environment and collective catering. Our study describes the first survey of *mcr-1* in ESBL-producing E. coli isolates from hospital cockroaches. Our results further highlight the possibility that *mcr-1* may enter humans via cockroach contamination and thereby threaten public health. Our results show that these cockroaches are an important reservoir of colistin-resistant E. coli and carriers of other additional risk genes such as *bla*_ESBL_, hence the importance of strengthening prevention strategies and of strictly respecting hygiene measures in order to control their distribution and spread in Tunisia.

## INTRODUCTION

The emergence of multidrug-resistant *Enterobacteriaceae* has become a major concern worldwide (1). For the past decade, *Enterobacteriaceae* genes (especially those of Escherichia coli and Klebsiella pneumoniae) have frequently been observed encoding ampicillin-hydrolyzing β-lactamases, leading to the use of third-generation cephalosporins (2). Third-generation cephalosporins are considered clinically important antimicrobials for the treatment of highly dangerous bacterial infections, due to their efficacy in a broad range of clinical treatments. In addition, the side effects of cephalosporins are less significant than those of penicillin antibiotics and other antibacterial agents (3, 4).

*Enterobacteriaceae* pursue various molecular strategies for development of resistance to β-lactams, and the resistance to various types of β-lactam antibiotics, including the extended-spectrum cephalosporins, is essentially induced by the production of extended-spectrum beta-lactamases (ESBLs) (5). The extended-spectrum β-lactamases are bacterial enzymes that inactivate β-lactam antibiotics. ESBLs can hydrolyze cephalosporins and monobactams but not cephamycins and carbapenems, conferring a high level of multidrug resistance (6).

In recent years, the plasmid-mediated *mcr-1* gene has been detected worldwide in human and animal samples (7, 8). In addition, cooccurrence of ESBL genes and *mcr-1* has been reported in *Enterobacteriaceae* (7, 9). A recent report showed that the coharboring of *mcr-1* and *bla*_CTX-M-1_ found on a conjugative IncHI2 type plasmid, together with other genes, confers multidrug resistance (10, 11). Consequently, antibiotic therapy has become limited for treating bacterial infections in both human and veterinary clinical settings (12).

Insects are known to be sources of transmission and spread of infectious diseases (13). Cockroaches play an important role as potential mechanical vectors in the transmission of pathogenic microorganisms; they are widely distributed in the environment, including houses, food industries, kitchens, and hospitals (14). Blattella germanica, Periplaneta americana, and Blatta orientalis are the most abundant cockroach species, and they are associated with human habitations (15, 16). They can be infected with different species of vertebrate pathogens under natural or *in vitro* conditions (13).

Cockroaches can carry medically important bacteria on the surface of their exoskeleton (e.g., legs, mouthparts) and in the digestive tract. Therefore, bacterial transmission can occur through regurgitation, defecation, or translocation from the exoskeleton. Furthermore, degrading cockroaches can contaminate the environment with bacteria resistant to antimicrobials, making these insects a threat to human health (17).

Moreover, the transmission and spread of multidrug-resistant *Enterobacteriaceae* in both hospital and community settings are generally related to poor hygiene, hand contact, and contaminated instruments (18, 19). Nevertheless, cockroaches which colonize these environments have been suggested as potential vectors of pathogenic antimicrobial-resistant bacteria, and they can have a great impact in the epidemiology of nosocomial infections, particularly those caused by strains of ESBL-producing *Enterobacteriaceae* (15, 20, 21).

**FIG 1:**
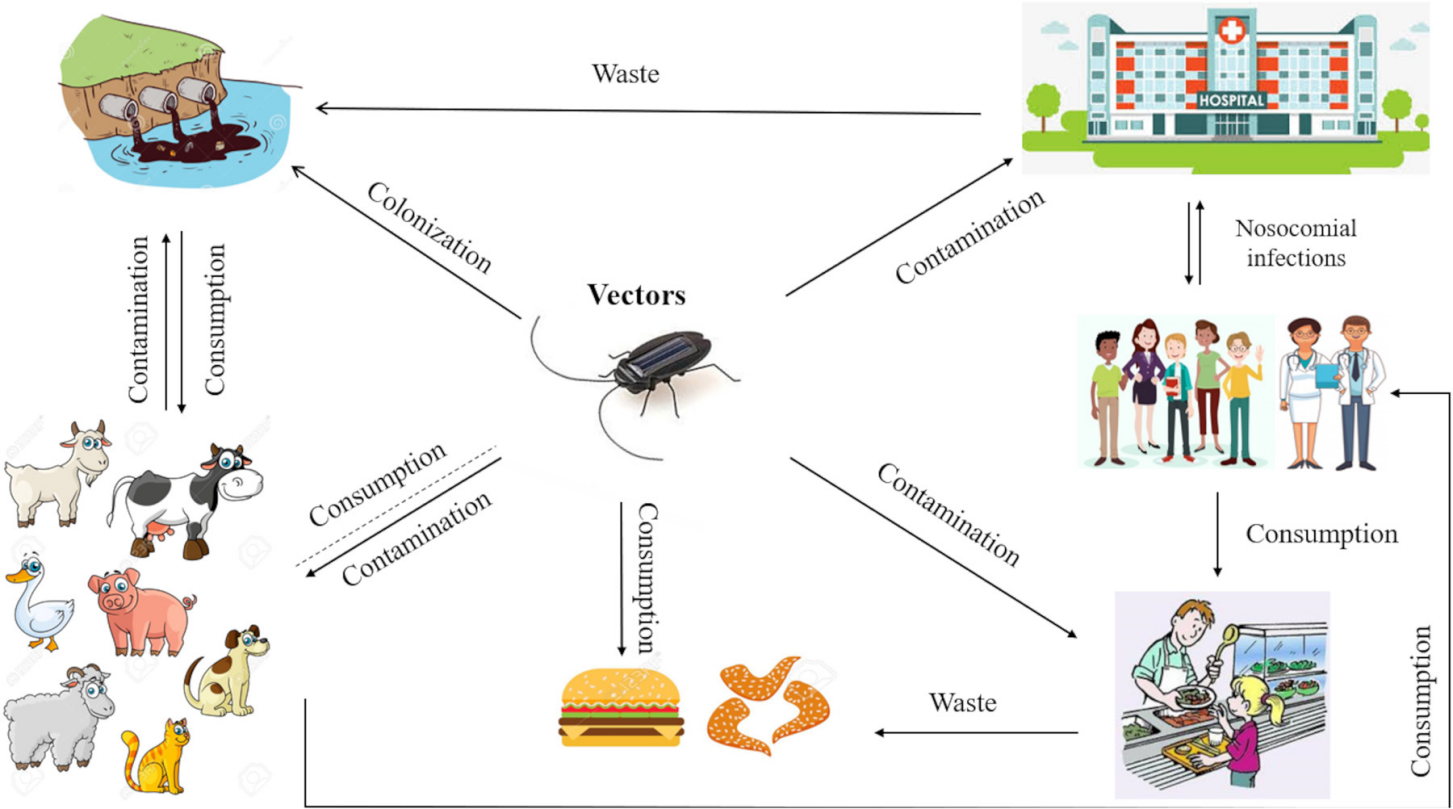
Antimicrobial resistance (AMR) interactions and transmission among different habitats.

Antimicrobial resistance requires a one-health, one-world approach (10, 22). In particular, resistant bacteria reside within humans, animals, food and the environment, and pathogens can be widely disseminated in agricultural and human waste (Fig. 1). Likewise, there are no barriers to the transmission of resistance genes across bacterial species and compartments (22, 23).

The aims of this study were first to evaluate the *Enterobacteriaceae* carrier rate on the external surface and gut homogenates of cockroaches and then to determine their antimicrobial susceptibility patterns, screen for ESBL-producing and colistin-resistant strains, and compare their molecular support in cockroaches collected from a collective catering, a hospital, and houses in Tunisia.

## RESULTS

### Bacterial isolation.

From July 2017 to June 2018, a total of 115 cockroaches were trapped in collective catering (*n* = 48), houses (*n* = 35), and a hospital (*n* = 32). Eighty-seven isolates from cockroaches were found to be P. americana, 21 were B. germanica, and 7 were B. orientalis.

From the 115 cockroaches trapped, 144 bacterial isolates were obtained. Table 1 illustrates the prevalence of *Enterobacteriaceae* isolated from different types of cockroaches trapped in different sites. E. coli (34.48%) was the predominant species from external surface and gut homogenates of cockroaches, followed by K. pneumoniae (31.72%) and Enterobacter cloacae (19.31%) (Table 1).

**TABLE 1:** *Enterobacteriaceae* isolates identified from external surface and gut homogenates of cockroaches trapped in different sites^*a*^

Bacterial isolate	No. and type of cockroach (*n* = 115) in bacteria:																			
	*P. americana* (*n* = 87)						*B. germanica* (*n *= 21)						*B. orientalis* (*n* = 7)							
	EH			GH			EH			GH			EH			GH				
	Hospital	CC	House	Hospital	CC	House	Hospital	CC	House	Hospital	CC	House	Hospital	CC	House	Hospital	CC	House		Total
E. coli	11	8	2	15	3	2	0	2	0	0	2	0	2	1	0	2	0	0		50
K. pneumoniae	2	7	3	4	7	5	0	10	0	0	8	0	0	0	0	0	0	0		46
E. cloacae	6	1	2	1	3	2	0	5	0	0	7	0	0	0	0	0	1	0		28
Serratia marcescens	0	0	0	3	0	2	0	0	0	0	0	0	0	0	0	0	0	0		5
Citrobacter freundii	0	1	0	0	1	0	0	0	0	0	2	0	0	0	0	0	0	0		4
Providencia rettgeri	0	1	0	0	2	0	0	0	0	0	0	0	0	0	0	0	0	0		3
Citrobacter gillenii	0	0	1	0	0	0	0	1	0	0	0	0	0	0	0	0	0	0		2
Citrobacter sedlakii	1	0	0	0	0	1	0	0	0	0	0	0	0	0	0	0	0	0		2
Serratia rubidaea	0	0	0	0	0	0	0	1	0	0	1	0	0	0	0	0	0	0		2
Pantoea calida	0	0	0	0	0	1	0	0	0	0	0	0	0	0	0	0	0	0		1
Leclercia adecarboxylata	0	0	1	0	0	0	0	0	0	0	0	0	0	0	0	0	0	0		1
																				
Total	20	18	9	23	16	13	0	19	0	0	20	0	2	1	0	2	1	0		144

a EH, external homogenate; GH, gut homogenate; CC, collective catering.

### Antimicrobial susceptibility testing.

Antibiotic sensitivity testing was performed for all isolates for 16 antibiotics. A high prevalence of resistance was found to amoxicillin (72.4%), amoxicillin-clavulanic acid (60.7%), and cephalothin (63.4%). Furthermore, 21.4% of strains were resistant to doxycycline and 20.7% to ceftriaxone and nitrofurantoin.

**FIG 2:**
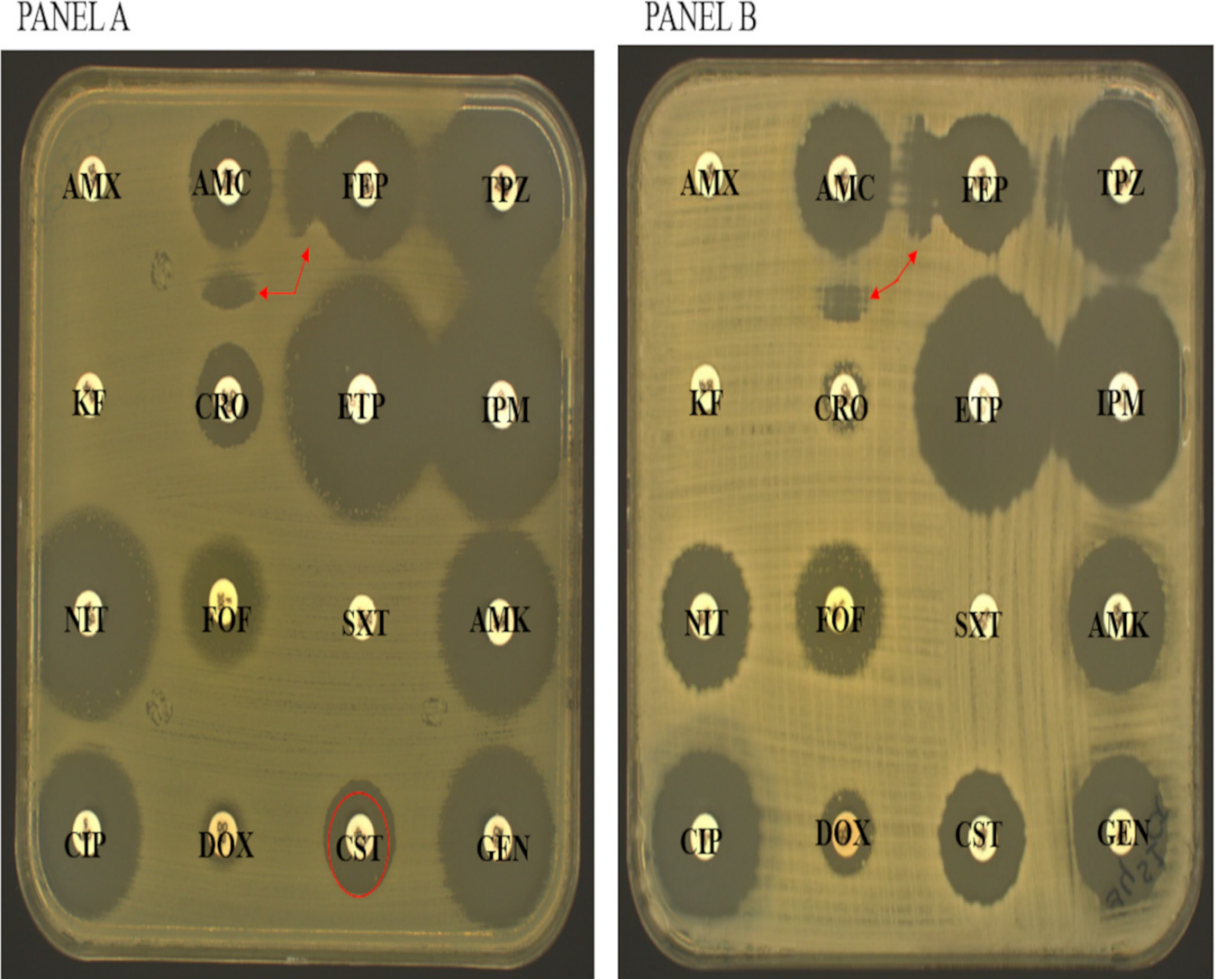
Examples of susceptibility testing plates. (A) Antibiogram by diffusion of E. coli (EC2) isolated from hospital expressing *bla*_CTX-M1_ and *mcr-1*. (B) Klebsiella pneumoniae isolated from collective catering expressing *bla*_CTX-M15_. AMX: amoxicillin, AMC: amoxicillin-clavulanic acid, TZP: piperacillin-tazobactam, KF: cephalothin, CRO: ceftriaxone, FEP: cefepime, ETP: ertapenem, IMP: imipenem, AMK: amikacin, GEN: gentamicin, CIP: ciprofloxacin, FOF: fosfomycin, NIT: nitrofurantoin, DOX: doxycycline, SXT: trimethoprim-sulfamethoxazole, CST: colistin.

Ceftriaxone-resistant isolates (30/144; 20.8%) were recovered from the different sites analyzed (16/30 in a hospital, 13/30 in collective catering, 1/30 in houses). Among the 30 ceftriaxone-resistant isolates, 22 had a positive ESBL-screening test (73.3%), including 17 E. coli and 5 K. pneumoniae isolates (Fig. 2B).

ESBL-positive isolates were also resistant to the non-beta-lactam antibiotics (Table 2), being resistant to trimethoprim-sulfamethoxazole (17/22; 77.2%), doxycycline (17/22; 77.2%), ciprofloxacin (16/22; 72.7%), and colistin (9/22; 40.9%), but all isolates showed susceptibility to carbapenems (ertapenem and imipenem).

**TABLE 2:** Characteristics and antibiotic resistance profile of strains harboring ESBL and *mcr-1* genes

Strain	Isolate source/location^*a*^	Bacteria species	Resistance profile^*b*^																MIC	Gene(s)	MLST
			AMX	AMC	TZP	KF	CRO	FEP	ETP	IMP	AMK	GEN	CIP	FOF	NIT	DOX	SXT	CST			
EC1	Hospital/GH	E. coli	R	I	S	R	R	R	S	S	S	S	I	S	S	R	R	R	16 μg/mL	*bla*_TEM-135_; *bla*_CTX-M1_; *mcr-1*	ST648
EC2	Hospital/GH	E. coli	R	I	S	R	R	I	S	S	S	S	I	S	S	R	R	R	8 μg/mL	*bla*_TEM-135_; *bla*_CTX-M1_; *mcr-1*	ST648
EC3	Hospital/GH	E. coli	R	I	S	R	R	R	S	S	S	S	I	S	S	R	R	R	16 μg/mL	*bla*_TEM-159_; *bla*_CTX-M1_; *mcr-1*	ST648
EC4	Hospital/GH	E. coli	R	I	S	R	R	I	S	S	S	S	I	S	S	R	R	R	8 μg/mL	*bla*_TEM-135_; *bla*_CTX-M1_; *mcr-1*	ST648
EC5	Hospital/EH	E. coli	R	I	S	R	R	R	S	S	S	S	I	S	S	R	R	R	8 μg/mL	*bla*_TEM-126_; *bla*_CTX-M1_; *mcr-1*	ST648
EC6	Hospital/GH	E. coli	R	I	S	R	R	I	S	S	S	S	I	S	S	R	R	R	16 μg/mL	*bla*_CTX-M1_; *mcr-1*	ST648
EC7	Hospital/GH	E. coli	R	I	S	R	R	I	S	S	S	S	I	S	S	R	R	R	16 μg/mL	*bla*_CTX-M1_; *mcr-1*	ST648
EC8	Hospital/EH	E. coli	S	I	S	R	R	I	S	S	S	S	S	S	S	S	S	S		*bla*_CTX-M15_	ST2914
EC9	Hospital/EH	E. coli	R	R	S	R	R	R	S	S	S	S	I	S	S	S	S	S		*bla*_CTX-M15_	ST2914
EC10	Hospital/GH	E. coli	R	I	S	R	R	I	S	S	S	S	I	S	S	R	R	R	8 μg/mL	*bla*_TEM-159_; *mcr-1*	ST648
EC11	Hospital/GH	E. coli	R	I	S	R	R	R	S	S	S	S	I	S	S	R	R	R	8 μg/mL	*bla*_TEM-1_; *mcr-1*	ST648
KP1	Hospital/GH	K. pneumoniae	R	I	S	R	R	R	S	S	S	R	S	S	S	R	R	S		*bla*_TEM-117_; *bla*_CTX-M15_	ST551
EC12	Collective catering/GH	E. coli	R	R	S	R	R	R	S	S	S	S	S	S	S	R	R	S		*bla*_TEM-128_; *bla*_CTX-M15_	ST8149
EC13	Collective catering/EH	E. coli	R	I	S	R	R	R	S	S	S	S	S	S	S	R	R	S		*bla*_CTX-M15_	ST8149
KP2	Collective catering/GH	K. pneumoniae	R	S	S	R	R	R	S	S	S	S	I	S	S	R	R	S		*bla*_CTX-M15_	ST2010
KP3	Collective catering/GH	K. pneumoniae	R	I	S	R	R	R	S	S	S	S	I	S	S	R	R	S		*bla*_CTX-M15_	ST2010
EC14	House/GH	E. coli	R	R	S	R	R	R	S	S	S	S	R	S	S	S	S	S		*bla*_CTX-M15_	ST155

*^a^*EH, external homogenate; GH, gut homogenate.

b MX, amoxicillin; AMC, amoxicillin-clavulanic acid; TZP, piperacillin-tazobactam; KF, cephalothin; CRO, ceftriaxone; FEP, cefepime; ETP, ertapenem; IMP, imipenem; AMK, amikacin; GEN, gentamicin; CIP, ciprofloxacin; FOF, fosfomycin; NIT, nitrofurantoin; DOX, doxycycline; SXT, trimethoprim-sulfamethoxazole; CST, colistin; R, resistant; I, intermediate; S, susceptible; MLST, multilocus sequence typing. Genes: CTX-M, especially *bla*_CTX-15_, conferring resistance to all the penicillins and cephalosporins and high-level resistance to other classes of antibiotics, especially fluoroquinolones and cotrimoxazole, but not carbapenems; TEM, conferring resistance to penicillin family antibiotics such as ampicillin and aminopenicillins (especially *bla*_TEM-1/135_); MCR-1, conferring resistance to colistin.

Furthermore, nine E. coli isolates were found to be resistant to colistin, with MICs between 8 and 16 μg/L. All isolates were resistant to amoxicillin, amoxicillin-clavulanic acid, cephalothin, ceftriaxone, cefepime, ciprofloxacin, doxycycline, and trimethoprim-sulfamethoxazole (Table 2 and Fig. 2A).

### Molecular detection of antibiotic resistance.

Beta-lactamase-encoding genes were detected in 17 strains, corresponding to an ESBL phenotype that is characterized by resistance to penicillins and cephalosporins. Of the 17 ESBL producers, PCR and nucleotide sequencing identified *bla*_CTX-M-15_ in 8 E. coli isolates, and 2 of these isolates coharbored *bla*_TEM-117/128_. Seven strains harbored the *bla*_CTX-M-1_ gene; among them, five strains coharbored *bla*_TEM-135/159/126_.

Two strains harbored *bla*_TEM-1/159_ genes. In view of the antibiogram results, 5 strains could express other β-lactamases that were not researched in our study (Table 2). None of the isolates harbored *bla*_KPC_, *bla*_VIM_, *bla*_NDM_, and *bla*_OXA-48_ genes.

In addition, colistin MIC of strains EC2, EC4, EC5, EC10, and EC11 was 8 μg/mL, and that of EC1, EC3, EC6, and EC7 was 16 μg/mL. Standard PCR results and sequencing analyses showed that all nine colistin-resistant E. coli isolates were positive for the *mcr-1* gene, and all the strains were detected in cockroaches from the hospital. These colistin-resistant E. coli isolates coharbored ESBL genes, including 5 strains expressing both *bla*_CTX-M1_ and *bla*_TEM_, 2 harboring only *bla*_CTX-M1_, and 2 possessing *bla*_TEM_ (Table 2). None of the isolates harbored *mcr-2/3/4/5/8* genes.

### MLST analysis.

According to the MLST analysis, 7 different STs (sequence types) were observed among the 19 sequenced ESBL strains (E. coli [*n* = 16] and K. pneumoniae [*n* = 3]). Among the known STs, the most common ST was ST648 (*n* = 9), followed by ST2914, ST8149, ST2010, and ST155 (2 isolates each), whereas one isolate each of ST23 and ST551 was found (Table 2). MLST analysis showed that the 9 E. coli isolates carrying the *mcr-1* gene were assigned to only one sequence type, ST648.

The accession numbers of E. coli and K. pneumoniae ST are available in Table S1.

## DISCUSSION

In our study, we found that cockroaches may play an important role as a potential vector of multidrug-resistant bacteria on their external surface and in their gut. In addition to the few reports describing the presence of ESBL-producing *Enterobacteriaceae* in cockroaches (21, 24–26), this study describes the first detection of the coharboring of *mcr-1* and *bla*_ESBL_ genes in nine E. coli strains of lineage ST648 from cockroaches isolated in the hospital.

The present study shows that cockroaches are potential vectors of multidrug-resistant *Enterobacteriaceae* in the hospital and collective catering environments (21). From the 115 cockroaches trapped, 144 bacterial isolates were obtained. E. coli (34.48%) was the predominant species from external and gut homogenates of cockroaches, followed by K. pneumoniae (31.72%) and E. cloacae (19.31%). *Enterobacteriaceae* are known as the essential cause of nosocomial and community infections, in particular pneumonia, urinary tract infection, respiratory tract infection, skin infections, septicemia, and gastroenteritis (17, 20). Drug sensitivity results also showed surprisingly high rates of resistance to some of the antibiotics. A high prevalence of resistance was found to amoxicillin (72.4%), amoxicillin-clavulanic acid (60.7%), and cephalothin (63.4%). Also, 21.4% of the strains were resistant to doxycycline, 20.7% to ceftriaxone and nitrofurantoin, and 6.25% to colistin, which is a last-line antibiotic for humans, especially for urinary tract infections (27).

Liu and colleagues (7) described for the first time the plasmid-borne gene *mcr-1* encoding resistance to colistin from animals, foodstuffs, and human beings in China. Subsequently, a series of variant *mcr* genes was identified, including *mcr-2* to *mcr-10* (28–30). In Tunisia, no study has described the presence of the *mcr-1* gene in hospitals. In contrast, the highest *mcr-1* prevalence was reported from animals (31–36).

Many studies have shown that ST648 is a developing and generalist lineage that lacks phylogeographic signals and clear host association. ST648 has been reported in human and companion animals in South America, the Middle East, and China. In addition, the combination of multidrug resistance and high-level virulence is the hallmark of ST648 and is similar to the international high-risk clonal lineage of ST131. ST648 became an epidemic vector for circulation/spread of the *mcr-1* colistin resistance gene in China (37–39).

In this study, the presence of beta-lactamase-encoding genes was detected in 17 strains, corresponding to an ESBL phenotype which is characterized by resistance to penicillins and cephalosporins. *bla*_CTX-M-15_ was the most dominant ESBL type (*n* = 8). It was found in four isolates from cockroaches collected from collective catering, typed as ST8149 or ST2010 (each *n* = 2, in E. coli and K. pneumoniae, respectively), those STs having been described in several studies in animal, food, and environmental isolates (40, 41). Three CTX-M15-producing isolates recovered from the hospital were typed as ST2914 (*n* = 2; E. coli), which has been reported in only one study in isolates of migratory birds in Pakistan (42), or ST551 (*n* = 1; K. pneumoniae), previously detected in human and environmental isolates (43, 44). Only one CTX-M15-producing isolate recovered from household cockroaches belonged to ST155. ST155 has been found in human clinical isolates, associated with ESBL phenotypes (45).

Consistent with previous studies showing that *bla*_CTX-M-15_ is amply present in Tunisia (46), our results suggest that this enzyme is encoded by the gene that mediates resistance to extended-spectrum cephalosporins in our strains. Furthermore, the isolation of ESBL-producing *Enterobacteriaceae* in household cockroaches is alarming, since the strains could easily spread and trigger a pandemic spread of the clones (24).

It is known that the transfer of resistance genes occurs by different genetic mechanisms, either by horizontal transfer of resistance genes located on different types of mobile DNA elements (transformation, conjugation, or transduction) or by mutations (point mutations, deletions, inversions) (47). ESBL-encoding genes can be transmitted between bacteria by mobile genetic elements, and cockroaches can provide a suitable environment for the exchange of resistance genes between different bacterial species. Therefore, effective prevention and control are needed to reduce nosocomial and foodborne bacterial infections. Cockroach infestation should be of serious concern, given the possible role of cockroaches as reservoirs of antibiotic-resistant bacteria.

Nine E. coli isolates were found to be resistant to colistin, with MICs between 8 and 16 μg/mL. All these strains also harbored the *mcr-1* gene and typed as ST648. Seven strains harbored *bla*_CTX-M-1_ genes, including five strains that coharbored *bla*_TEM-135/159/126_. Two strains harbored *bla*_TEM-1/159_. All isolates were resistant to amoxicillin, amoxicillin-clavulanic acid, cephalosporins, doxycycline, and trimethoprim-sulfamethoxazole, and all them were collected from the same hospital. Cockroaches are frequently present in hospital environment; humidity and water damage are important factors of proliferation of insects (48). In general, high relative humidity and low temperature provide the best chances for cockroaches to survive (16). Most strains were isolated from the gut, which could be explained by the great adaptation of these organisms to the intestinal environment (21).

ESBL and *mcr* genes were frequently detected in several species of *Enterobacteriaceae*, including K. pneumoniae and E. coli, which are responsible for nosocomial infections in humans and are of particular concern for multidrug resistance (9, 35, 49). Our study describes the first detection of the coharboring of *mcr-1* and *bla*_ESBL_ from cockroaches in nine E. coli strains of lineage ST648 isolated from a hospital. This E. coli lineage has emerged as a pandemic clone, and the association between ST648 and ESBL genes has been reported in companion animals (50–52). The presence of the ESBLs and *mcr-1* genes in E. coli strains of lineage ST648 in cockroaches is a public health concern, since it could indicate an important and silent diffusion of this clinically significant clone in the human-animal environment interface (51).

The results obtained in several studies, including ours, should better explain a world in which human health, animal health, and the environment are directly or indirectly linked (24, 53, 54).

Cockroaches ingest fluids that can be contaminated with multidrug-resistant bacteria. These bacteria multiply in the digestive tract and are then transferred to the intestine or are regurgitated. As cockroaches share their habitat with animals and humans, transmission of antimicrobial resistance is, therefore, possible (55). Therefore, since cockroaches are a source of multidrug-resistant pathogens and a risk factor for dissemination of these bacteria to humans and/or other animal species in contact with humans, methods to control cockroaches are needed to decrease the spread of nosocomial and foodborne bacteria (16). Surveillance of hospital infections should be complemented by monitoring disinfectants used in the hospital as well as evaluation of infestation of the hospital environment and resistance of cockroaches to biocides, and all water points in intensive care units must be closed (56).

We conducted the first survey of *mcr-1* in ESBL-producing E. coli isolates from hospital cockroaches. Our results further highlight the possibility that *mcr-1* may enter humans via cockroach contamination and thereby threaten public health. Our results show that these cockroaches are an important reservoir of colistin-resistant E. coli and carriers of other additional risk genes such as *bla*_ESBL_, hence the importance of strengthening prevention strategies and of strictly respecting hygiene measures in order to control their distribution and spread in Tunisia.

## MATERIALS AND METHODS

### Collection and identification of cockroaches.

A total of 115 adult cockroaches were collected from July 2017 to June 2018 in Tunisia.

Forty-eight cockroaches were obtained from collective catering, 35 cockroaches were trapped from different parts of houses (kitchens, garden, bathrooms, and toilets), and the remaining 32 were obtained from different parts of a hospital (kitchen and emergency department).

Cockroaches were collected using sterile and separate flasks and transported to our laboratory for bacteriological analysis. After immobilization by freezing at 0°C for 5 min, species identification was done according to the method of Harwood et al. (57).

To wash the external body surface, sterile normal saline (3 mL) was added to each flask and the cockroaches were vigorously washed and transferred to secondary sterile flasks. After washing and decontamination with 70% alcohol, the gut of the cockroaches was dissected and crushed aseptically in a sterile pestle and mortar. The gut was then maintained in a 3 mL sterile physiological saline solution for 5 min to produce a homogenate sample.

### Bacterial isolation.

After the external and gut homogenates were prepared and placed in test tubes containing normal saline solution, each sample (1 mL) was preenriched for 24 h at 37°C in buffered peptone water (BPW) and buffered peptone water supplemented with cefotaxime (CTX; 1 mg/L) to isolate cefotaxime-resistant bacteria.

After overnight incubation, the samples were inoculated on MacConkey agar (MCA) and MacConkey agar with 1 mg/L of cefotaxime and then incubated for 24 h at 37°C. Colonies showing *Enterobacteriaceae* morphology were recovered and identified by matrix-assisted laser desorption ionization–time of flight mass spectrometry.

### Antimicrobial susceptibility testing and ESBL identification.

Susceptibility testing was performed using a standard disk diffusion technique according to the recommendations of the Antibiogram Committee of the French Society for Microbiology, 2019. The antimicrobial agents tested included amoxicillin, amoxicillin-clavulanic acid, piperacillin-tazobactam, cephalothin, ceftriaxone, cefepime, ertapenem, imipenem, amikacin, gentamicin, ciprofloxacin, fosfomycin, nitrofurantoin, doxycycline, trimethoprim-sulfamethoxazole, and colistin.

After overnight incubation, the diameter of the zone of inhibition around the discs was measured and interpreted as susceptible, intermediate, and resistant.

To screen for the production of ESBLs, one cefotaxime-resistant isolate obtained per sample was selected and screened for the ESBL phenotype by the double-disk synergy test (DDST).

The detection of colistin-resistant isolates was confirmed for all isolates showing an intermediate and resistant phenotype by disk diffusion by the determination of the MICs of colistin with the UMIC test (Biocentric, Bandol, France). Otherwise, the MICs were determined using Etest strips (bioMérieux, Marcy l’Etoile, France) for the isolates showing carbapenem-non-susceptibility detected with ertapenem and imipenem. The results were interpreted according to CA-SFM/EUCAST (2019) breakpoints.

### Characterization of beta-lactamase and colistin resistance genes.

After DNA extraction for all samples by the boiling method, the presence of resistance genes was screened using real-time PCR, with specific primers (ESBL-encoding genes: *bla*_CTX-M_, *bla*_SHV_, *bla*_TEM_; carbapenem resistance-encoding genes: *bla*_KPC_, *bla*_NDM_, *bla*_VIM_, and *bla*_OXA-48_) (58).

All colistin-resistant isolates were screened for the presence of *mcr-1*, *mcr-2*, *mcr-3*, *mcr-4*, *mcr-5*, and *mcr-8* genes (59).

The positive strains detected by real-time PCR were submitted to conventional PCR to confirm the previous results. Subsequently, products were purified and sequenced using the BigDye terminator chemistry on an ABI 3130XL automated sequencer (Thermo Fisher Scientific, Waltham, MA, USA). The obtained sequences were analyzed using ChromasPro and compared to the ARG-ANNOT database and NCBI GenBank database (https://www.ncbi.nlm.nih.gov/).

### Phylogenetic analysis.

The determination of a genetic relationship among isolates was done by multilocus sequence typing (MLST) using the seven housekeeping genes. Each gene was amplified and sequenced for each E. coli (*adk*, *fumC*, *gyr*, *icd*, *mdh*, *purA*, and *recA*) and K. pneumoniae (*gapA*, *infB*, *mdh*, *pgi*, *phoE*, *rpoB*, *tonB*) strain, and then the sequence types (ST) were assigned in accordance with the online database (https://bigsdb.pasteur.fr/) for E. coli strains; for the strains of K. pneumoniae, we used https://pubmlst.org/.

### Data availability.

The accessions numbers of ST found in our study are available in Table S1.

The MLST sequences are available on MLST web database (https://bigsdb.pasteur.fr/) for K. pneumoniae and on https://pubmlst.org/ for E. coli strains.
