# Supplementary material for: First Report of Extended-Spectrum β-Lactamase (blaCTX-M1) and Colistin Resistance Gene mcr-1 in E. coli of Lineage ST648 from Cockroaches in Tunisia
Source: Microbiol Spectr. 2022 Mar 1;10(2):e00036-21. doi: 10.1128/spectrum.00036-21 (PMC9045256; doi:10.1128/spectrum.00036-21)
Supplement: SUPPLEMENTAL FILE 1 — Supplemental material. Download SPECTRUM00036-21_Supp_1_seq3.pdf, PDF file, 0.1 MB [file spectrum00036-21_supp_1_seq3.pdf]

Table S1: Allelic combinations of sequence types (ST) and their Genbank accession numbers

Strains	Sequence Types	Combinations of alleles						
E. coli		***adk***	***fumC***	***gurB***	***icd***	***mdh***	***purA***	***recA***
	**ST648**	92	4	87	96	70	58	2
	Genbank accession number	MZ346383.1	MW519917.1	MF381225.1	MF381234.1	MH561342.1	MZ346152.1	KM072943.1
	**ST8149**	64	11	5	8	693	8	35
	Genbank accession number	LC187169.1	MW557688.1	MW519952.1	MW557701.1	MN315061.1	KM072796.1	AB648888.1
	**ST2914**	34	36	207	87	67	16	4
	Genbank accession number	KF914279.1	MH561275.1	MG194495.1	LC187109.1	MG204771.1	LC187111.1	MH561375.1
	**ST155**	6	4	14	16	24	8	14
	Genbank accession number	MW519923.1	MW519917.1	MN529017.1	MW557695.1	MN315063.1	KM072796.1	MN315096.1
	**ST23**	6	4	12	1	20	13	7
	Genbank accession number	MW519923.1	MW519917.1	MN315005.1	MW519943.1	MN529105.1	MN315064.1	OU342919.1
K. pneumoniae		***gapA***	***infB***	***mdh***	***pgi***	***phoE***	***rpoB***	***tonB***
	**ST2010**	17	55	39	20	243	18	156
	Genbank accession number	HG933781.1	LC049012.1	AM051118.1	LC049121.1	KT270588.1	MF345933.1	FJ483747.1
	**ST551**	3	1	1	1	9	4	135
	Genbank accession number	LT615348.1	AJ227992.1	AJ890392.1	AJ890402.1	AJ890421.1	MT882043.1	AJ890491.1
